# Cancer-associated fibroblasts in hematologic malignancies: elucidating roles and spotlighting therapeutic targets

**DOI:** 10.3389/fonc.2023.1193978

**Published:** 2023-09-06

**Authors:** Ziyang Ding, Run Shi, Weikang Hu, Lei Tian, Rong Sun, Yang Wu, Xiaoyan Zhang

**Affiliations:** ^1^ The Second Clinical School of Nanjing Medical University, Nanjing, China; ^2^ Department of Oncology, The First Affiliated Hospital of Nanjing Medical University, Nanjing, China; ^3^ Pancreas Center, The First Affiliated Hospital of Nanjing Medical University, Nanjing, China; ^4^ Department of Radiation Oncology, Jinling Hospital, Nanjing, China; ^5^ Department of Hematology, Tongren Hospital, Shanghai Jiao Tong University School of Medicine, Shanghai, China

**Keywords:** cancer associated fibroblast (CAF), hematologic malignancies, crosstalk, chemoresistance, therapeutic target

## Abstract

Hematologic malignancies comprise a diverse range of blood, bone marrow, and organ-related disorders that present significant challenges due to drug resistance, relapse, and treatment failure. Cancer-associated fibroblasts (CAFs) represent a critical component of the tumor microenvironment (TME) and have recently emerged as potential therapeutic targets. In this comprehensive review, we summarize the latest findings on the roles of CAFs in various hematologic malignancies, including acute leukemia, multiple myeloma, chronic lymphocytic leukemia, myeloproliferative neoplasms, and lymphoma. We also explore their involvement in tumor progression, drug resistance, and the various signaling pathways implicated in their activation and function. While the underlying mechanisms and the existence of multiple CAF subtypes pose challenges, targeting CAFs and their associated pathways offers a promising avenue for the development of innovative treatments to improve patient outcomes in hematologic malignancies.

## Introduction

Hematologic malignancies encompass a diverse array of blood, bone marrow, and organ-related disorders. Presently, leukemias and lymphomas can be treated using drugs or drug combinations, such as chemotherapy, targeted therapies, immunotherapy, immune checkpoint inhibitors, and chimeric antigen receptor-T (CAR-T) cells. These treatments have significantly enhanced patient prognoses. However, emerging drug resistance poses a major challenge, leading to relapse and treatment failure (1).

CAFs constitute the largest proportion of stromal cells in the tumor microenvironment (TME) (2). The origin of CAFs remains a subject of debate, with fibroblasts and mesenchymal stem cells (MSCs) from bone marrow (BM) and adipose tissue reservoirs believed to be their primary source (3). No specific markers exist for CAFs, although elevated alpha-smooth muscle actin (αSMA) expression is considered indicative of activated CAFs (4). Exhibiting enhanced proliferative and migratory capabilities, CAFs significantly influence tumor progression (5). Numerous studies have established the critical role of CAFs in solid tumors such as pancreatic, breast, colon, gastric, and liver malignancies (6–10). Further research has also explored targeting mechanisms like the TGFβ signaling pathway and the JAK/STAT signaling pathway (11, 12). The significance of CAFs in tumor progression and drug resistance is increasingly acknowledged, making them a focal point of recent research. Promisingly, several CAF-targeting therapies have entered clinical trials (5, 13).

**Figure 1:**
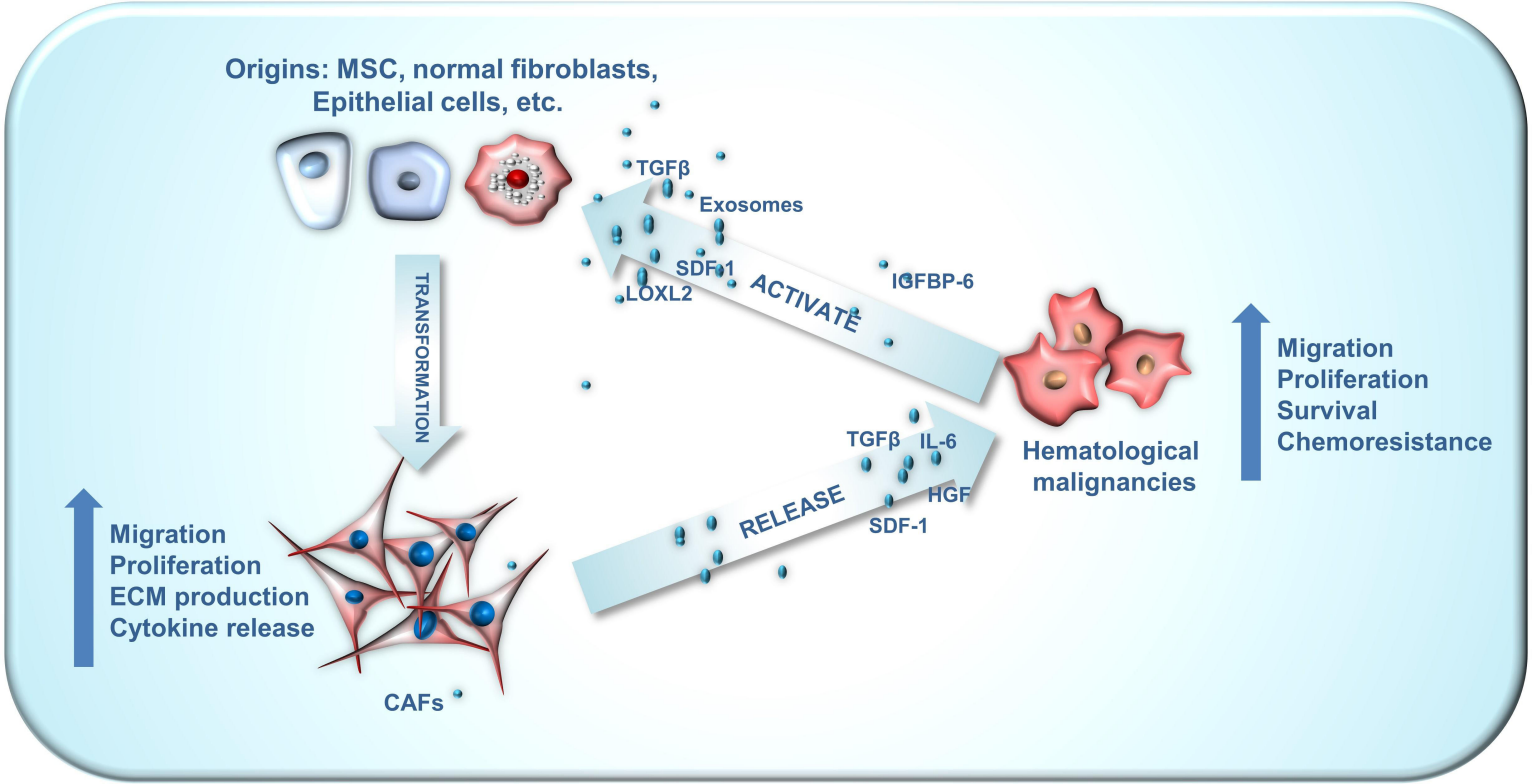
The crosstalk between CAF origins, CAFs and hematological malignancies. CAFs can emanate from a wide array of origins, encompassing mesenchymal stem cells, normal fibroblasts, myofibroblasts, endothelial cells, adipocyte pericytes, monocytes and macrophages, each exhibiting distinct phenotypes. The crosstalk between CAFs and hematological malignancies plays an important role in the development of blood cancer. Hematological malignancies are capable of facilitating the conversion of these diverse CAF origins into activated CAFs via numerous paracrine pathways. Subsequently, these activated CAFs can enhance the malignant phenotype of hematological malignancies through paracrine routes.

The intricate interplay between CAFs and cancer cells is crucial for their interaction and is evident in hematologic tumors as well (**Figure 1**). For instance, bone marrow stromal cells can adopt CAF phenotypes, with the latter secreting various cytokines to stimulate tumor cell growth, infiltration, and endosteal niche reconstruction. Concurrently, TME remodeling provides tumor stem cells additional time for clonal reproduction, resulting in the continuous emergence of new genetic mutations that drive disease progression. This CAF-mediated remodeling also contributes to drug resistance, relapse, and tumor cell progression. In this article, we provide a comprehensive review of recent literature and summarize the roles of CAFs in hematologic tumors, as well as their potential value in disease treatment.

## CAFs in acute leukemia (AL)

AL is characterized by the abnormal differentiation and proliferation of hematopoietic stem cells, which impedes normal hematopoiesis (14). Zhai et al. found that the presence of abundant reticulin fibers was associated with poor outcomes in acute myeloid leukemia (AML) (15). Their study showed that CAFs expressing elevated levels of FSP1, αSMA, or FAP protein were extensively distributed within the bone marrow (BM) of AML patients. They also proposed that CAFs could potentially shield leukemia cell lines (THP-1/K-562) from chemotherapy (15). By targeting growth differentiation factor-15 (GDF15) or suppressing GDF15 expression, the sensitivity of leukemic cells to chemotherapy increased, suggesting that GDF15 secretion by CAFs may play a crucial role in mediating the chemoprotective effects of CAFs (15).

Pan et al. carried out a series of investigations on CAFs in B-cell acute lymphoblastic leukemia (B-ALL) (16). They discovered that in newly diagnosed and relapsed B-ALL patients, bone marrow mononuclear cells had a higher percentage of CAF markers αSMA and FAP (16). Additionally, when BM-MSCs were co-cultured with leukemia cells, they adopted a CAF phenotype, which led to increased production of tumor-promoting growth factors and reduced daunorubicin-induced damage to B-ALL cells (16). Notably, while the chemoprotective effects of MSCs and CAFs on B-ALL were somewhat similar, CAFs proved to be more effective than MSCs in promoting the aggressiveness of B-ALL cells (16).

Subsequent research indicated that the overexpression of TGF-β plays a critical role in promoting the differentiation of BM-MSCs into CAFs, which may be dependent on the SDF-1/CXCR4 pathway (16, 17). The TGF-β receptor inhibitor LY2109761 and the CXCR4 antagonist AMD3100 both reduce CAF activation, offering a novel approach for chemotherapeutic regimens in AL (16–18). Li et al. isolated and cultured the first fibroblast tumor cell line, HXWMF-1, derived from CAFs in a 6-year-old B-ALL patient. They found compelling evidence that leukemic cells could potentially induce the malignant transformation of CAFs in a BALB/c nude mouse model (19).

## CAFs in multiple myeloma (MM)

MM is a disorder characterized by malignant plasma cell proliferation. The TME plays a substantial role in MM pathophysiology by secreting various cytokines that promote plasma cell survival, proliferation, and treatment resistance (20). Notably, the expression of CAF markers (FSP1, αSMA, FAP) in the bone marrow (BM) of patients with active MM was significantly higher (21). MM cells were found to induce CAF proliferation and enhance MM cell adhesion, proliferation, and apoptosis inhibition (21). The interaction between the two may be mediated through the SDF-1α/CXCR4 axis and integrins (21). Ciavarella et al. discovered that the activation levels of CAFs in MM patients at different clinical stages correlated with the expression of the fibrinolytic system (22). Compared to patients in the quiescent phase, CAFs in active MM patients exhibited higher transcriptional levels of u-PAR and u-PA. Selectively silencing u-PAR significantly suppressed CAF phenotype and function (22). Meanwhile, Kanehira et al. demonstrated that lysophosphatidic acid receptors 1 and 3 influenced the transition of MSCs to CAF differentiation, resulting in distinct outcomes (23).

Several targeted therapies for MM have emerged, but most have encountered drug resistance. For example, bortezomib, the first protease inhibitor approved by the FDA for MM treatment, has demonstrated limited efficacy in most patients due to the development of drug resistance (24). Several studies have investigated the vital role CAFs play in this issue. *In vitro* experiments indicated that CAFs from bortezomib-resistant patients inhibited bortezomib-induced apoptosis in MM cells. It is well-known that cellular autophagy contributes to drug resistance. When bortezomib-resistant CAFs are exposed to bortezomib, the autocrine TGF-pathway, which fosters autophagy, may become activated. Conversely, using TβR-I/II inhibitors to block Smad2/3 and autophagic pathways may help counteract MM resistance (25). CAR-T treatments targeting BCMA can detect and eradicate malignant plasma cells in MM patients, making them a promising therapeutic option. A study by Sakemura et al. revealed through ex vivo experiments that MM-CAFs inhibited antigen-specific proliferation of BCMA CAR-T cells via TGF-β secretion, consequently dampening their anti-myeloma activity (26). Simultaneously, targeting both MM cells and their CAFs with CAR-T cells reduced drug resistance development and slowed tumor progression, suggesting a new treatment approach (26).

## CAFs in chronic lymphocytic leukemia (CLL)

CLL is a cancer characterized by the uncontrolled growth of mature lymphocytes in the blood, bone marrow, lymph nodes, and spleen (27). In the context of CLL, CAFs play a critical role in disease progression and interaction with the tumor microenvironment. CLL cells have the ability to activate the AKT pathway and stimulate the proliferation of MSCs via platelet-derived growth factor (PDGF) receptors (28). Furthermore, both bone marrow-derived MSCs and endothelial cells (ECs) can adopt a CAF phenotype when exposed to CLL-derived exosomes (29). These exosomes contain various molecular signals that can influence the behavior of recipient cells. Recent research has shown that CLL cells can trigger the transformation of BM-MSCs into CAFs by releasing exosomes containing miR-146a, which in turn inhibits USP16 (30). Additionally, a significant presence of αSMA(+) stromal cells was identified in infiltrating lymph nodes, further confirming the existence of numerous CAFs in CLL patients (29). CAFs play a crucial role in shaping the CLL microenvironment by influencing various immune cell functions. They release cytokines and chemokines that contribute to T cell and myeloid cell immunosuppression, and activate the AKT and NF-κB pathways, all of which promote tumor progression (31).

## CAFs in myeloproliferative neoplasms (MPNs)

MPNs are malignant diseases characterized by excessive proliferation within the myeloid lineage, and αSMA, a CAF marker, is significantly elevated in MPN patients (32). Research suggests that αSMA expression levels influence the self-renewal and differentiation potential of MSCs, indicating a possible connection between αSMA expression and MPN development and prognosis (33). Primary myelofibrosis (PMF) is an MPN subtype characterized by progressive myelofibrosis. The development of myelofibrotic processes in PMF is currently believed to be associated with excessive stimulation of MSCs by growth factors (34).

In MPNs, lysyl oxidase (LOX), a stromal cross-linking protein, contributes to increased bone marrow stromal deposition. The use of a LOX inhibitor (BAPN) to decrease reticulin fibers supports LOX’s role in myelofibrosis development (35). LOXL2 expression is found to be elevated in MPN patients, especially those with PMF (36). Higher levels of LOXL2 may contribute to MPN progression by modulating the function of peripheral stromal cells that display a cancer-associated fibroblast phenotype (36). Furthermore, LOXL2 is considered a key factor in driving the differentiation of mesenchymal stem cells (MSCs) into CAFs (37). These discoveries provide novel perspectives for targeted MPN treatments. Simtuzumab, a monoclonal antibody that inhibits LOXL2, is currently being tested in phase II clinical trials (38).

In PMF patients, there is a significant expansion of clonal tumorigenic fibroblasts, a particular type of CAFs, which are functionally different from normal fibroblasts. This difference may be associated with JAK2 signaling, and these fibroblasts contribute to the progression of myelofibrosis (34). On the other hand, the fibroblast differentiation inhibitor SAP (PRM-151) substantially increases the survival rate of NSG mice transplanted with PMF bone marrow cells and reduces the development of myelofibrosis (34). Longhitano et al. found that exposure to IGFBP-6 leads to an increased expression of CAF markers (αSMA, FAP, TGF-β) in HS5 cells. Their research suggests that IGFBP-6 triggers the differentiation of MSCs into CAFs and indicates a connection between the IGFBP-6/SHH/TLR4 axis and alterations in the PMF microenvironment. This offers new perspectives on the pathogenesis of fibrosis in PMF patients (39).

## CAFs in lymphoma

Lymphoma is the most prevalent hematologic malignancy, divided mainly into non-Hodgkin’s lymphoma (90%) and Hodgkin’s lymphoma (10%) (40). CAF-like cells and their precursors are present in secondary lymphoid organs (SLOs) before lymphoma onset, playing a crucial role in the progression of malignancies. For example, fibroblastic reticular cells (FRCs) form the structural foundation of SLOs and are essential for organ development, T and B cell compartmentalization, and adaptive immune response involvement. This provides a supportive microenvironment for the proliferation of malignant B cells (41). Numerous studies indicate that CAFs can aid lymphocyte survival by enhancing glycolysis (42, 43). Metabolic analyses have shown that elevated concentrations of CAF-secreted pyruvate decrease intracellular ROS production in primary lymphoma cells, augment tumor cell dependence on the citric acid cycle, and boost tumor cell survival (44). Furthermore, CAFs modulate the expression of the pyrimidine transporter protein ENT2 in tumor cells by secreting exosomes containing miR-4717-5p, resulting in chemoresistance (43).

Diffuse large B-cell lymphoma (DLBCL), the most prevalent lymphoma type, triggers the activation process of CAFs. Activated CAFs display a compensatory suppressive response by increasing PD-L1 expression and reducing the lytic-killing activity of CD8 T cells against tumor cells (45, 46). These findings offer a fresh perspective on the disease’s initiation. Two CAF subtypes have been identified in adult T-cell leukemia/lymphoma (ATLL): CAFs/EGR_high_ and CAFs/EGR_low_. CAFs in ATLL were found to significantly contribute to CD4 T-cell proliferation

(47). Additionally, CAF/EGR_high_ influences CD8 and NKT cell expansion through EGFR (47). These findings suggest potential avenues for targeted therapy.

## CAF-related targets and pathways in hematologic malignancies

CAFs have emerged as critical contributors to hematologic malignancies, influencing tumor progression and drug resistance. While the underlying mechanisms of CAF activation and function in hematologic malignancies are not yet fully understood, recent research has highlighted several potential therapeutic targets and pathways (48). Targeting CAFs and their associated pathways could provide an innovative approach to treating hematologic malignancies and enhancing patient outcomes. As research into the interplay between CAFs and hematologic malignancies continues, there is a promising prospect for developing novel treatments that target CAFs and improve clinical outcomes for patients.

**Table 1** provides a summary of the latest research advancements in CAF-related targets and pathways within the context of hematologic malignancies.

**Table 1:** Passways/targets associated with CAFs in hematological malignancies.

Target/Pathway	Hematologic Malignancy	Discovery/Advancement
TGF-β signaling pathway	AL, MM	Overexpression of TGF-β induces differentiation of BM-MSCs into CAFs; TGF-β receptor inhibitors reduce CAF activation (16–18)
JAK/STAT signaling pathway	AL, PMF	Clonal tumorigenic fibroblasts in PMF patients have functional differences associated with JAK2 signaling; targeting the JAK/STAT pathway may provide a new approach for AL treatment (34)
SDF-1/CXCR4 pathway	AL, MM	The activation of BM-MSCs into CAFs is dependent on the SDF-1/CXCR4 pathway; CXCR4 antagonists may reduce CAF activation (16, 21)
Autophagy	MM	Bortezomib-resistant CAFs may foster drug resistance through the autocrine TGF-β pathway and autophagy; blocking the TGF-β pathway may counteract drug resistance (25)
LOX/LOXL2	MPN	LOXL2 drives MSC differentiation into CAFs and contributes to MPN progression by modulating peripheral stromal cells; LOX inhibitors and LOXL2 inhibitors are being tested for targeted MPN treatment (35–37).
PD-1/PD-L1 pathway	DLBCL	Activated CAFs increase PD-L1 expression and reduce CD8 T cell lytic-killing activity against tumor cells (45, 46)
SHH/TLR4 axis	MPN	IGFBP-6 may trigger the differentiation of MSCs into CAFs via the IGFBP-6/SHH/TLR4 axis (39)
Exosomal miR-4717-5p	Lymphoma	CAFs modulate ENT2 expression in tumor cells by secreting exosomes containing miR-4717-5p, resulting in chemoresistance (43)

## Discussion

In recent years, the understanding of cancer biology has expanded, leading to the identification of various cellular and molecular players involved in tumor progression. One such player is CAFs, which have been implicated in the progression of solid tumors. However, their role in blood cancers remains underexplored. In this discussion, we will delve into the potential involvement of CAFs in blood cancers and evaluate their suitability as a promising therapeutic target.

To begin, it is essential to understand the role of CAFs in the tumor microenvironment. CAFs are key stromal cells that modulate the extracellular matrix, support angiogenesis, and produce a myriad of growth factors and cytokines. These actions contribute to the tumor-promoting milieu, ultimately enhancing cancer cell survival, proliferation, and metastasis. Given their critical role in solid tumors, it is plausible to assume that CAFs may have similar functions in blood cancers.

Blood cancers, such as leukemia, lymphoma, and myeloma, arise from the malignant transformation of cells in the blood, bone marrow, or lymphatic system. Although these cancers lack the solid tumor architecture, they still interact with the surrounding microenvironment, which may include CAFs. For instance, interactions between leukemia cells and bone marrow stromal cells, including fibroblasts, have been reported to support leukemia cell survival and contribute to therapeutic resistance. This suggests that CAFs could be critical players in the pathogenesis of blood cancers.

Targeting CAFs as a therapeutic strategy in blood cancers may have several advantages. First, as stromal cells, CAFs are genetically more stable than cancer cells, making them less likely to develop resistance to targeted therapies. Second, by disrupting the crosstalk between CAFs and cancer cells, the tumor-promoting microenvironment could be altered, potentially enhancing the efficacy of existing treatments. Finally, targeting CAFs may have a synergistic effect when combined with other therapies, leading to improved clinical outcomes.

However, it is important to consider the challenges and limitations associated with targeting CAFs in blood cancers. One of the primary challenges lies in the heterogeneity of CAFs, as they can originate from various cell types and exhibit diverse phenotypes and functions. This complexity may hinder the development of specific CAF-targeted therapies and could necessitate the identification of common signaling pathways or markers that can be targeted across different CAF subpopulations (4, 5). Cancer boasts a multifaceted biological composition and structure, encompassing cancerous cells, stromal cells, and the extracellular matrix (49). Historically, the majority of treatments have primarily aimed at cancer cells themselves (49). However, recent research has shed light on the significant influence the TME has on the behavior of cancer cells and their response to therapies (49, 50). Notably, CAFs, which constitute the most prevalent type of stromal cells within the TME, play a crucial yet understated role in the inception, progression, and metastasis of cancer (49). Consequently, focusing research on TME and CAF markers has emerged as a pivotal component of innovative strategies for the design and discovery of next-generation cancer drugs (49). However, unlike the case with solid tumors, the study of the tumor microenvironment and CAF markers in fibroblasts associated with hematological malignancies is still in its early stages (49, 51, 52). This remains an important area for future exploration and research.

Another challenge is the potential for off-target effects, given that CAFs share similarities with normal fibroblasts. Developing therapies that selectively target CAFs without affecting healthy fibroblasts is essential to minimize adverse side effects. Furthermore, the dynamic nature of the tumor microenvironment and the reciprocal interactions between CAFs and cancer cells may result in compensatory mechanisms that limit the efficacy of CAF-targeted therapies. Therefore, understanding the molecular mechanisms underlying these interactions is crucial for the development of effective treatment strategies (53).

In light of these challenges, future research should focus on elucidating the molecular and cellular mechanisms that govern CAFs’ involvement in blood cancers. High-throughput screening technologies, such as single-cell RNA sequencing, could provide valuable insights into the heterogeneity of CAF populations and identify potential therapeutic targets (54–56). Additionally, the development of advanced *in vitro* and *in vivo* models that more closely mimic the tumor microenvironment will be essential for evaluating the safety and efficacy of novel CAF-targeted therapies. Moreover, the potential synergistic effects of combining CAF-targeted therapies with other treatment modalities, such as chemotherapy, immunotherapy, and targeted therapies, should be investigated. This combinatorial approach may help overcome potential resistance mechanisms and improve clinical outcomes for patients with blood cancers.

In summary, the targeting of CAFs in blood cancers presents a promising therapeutic strategy, but it is not without challenges. Future research should address the limitations and obstacles associated with CAF-targeted therapies and explore the potential benefits of combining these treatments with existing therapies. By deepening our understanding of CAFs’ role in blood cancers and overcoming the hurdles associated with their targeting, we may be able to unlock new, more effective treatment options for patients suffering from these malignancies.

## Author contributions

Investigating and writing the manuscript, ZYD and RUS. Editing and revising, WKH and ROS. Literature reading and review, LT. Project administration, YW and XYZ. All authors contributed to the article and approved the submitted version.
